# EHA Endorsement of the Global Guideline for the Diagnosis and Management of Rare Yeast Infections: An Initiative of the European Confederation of Medical Mycology in Cooperation With the International Society for Human and Animal Mycology and American Society for Microbiology

**DOI:** 10.1097/HS9.0000000000000644

**Published:** 2021-09-30

**Authors:** Jannik Stemler, Ľuboš Drgoňa

**Affiliations:** 1 Center for Integrated Oncology Aachen Bonn Cologne Duesseldorf (CIO ABCD), European Diamond Excellence Center for Medical Mycology (ECMM), Department I of Internal Medicine, Faculty of Medicine and University Hospital of Cologne, University of Cologne, Germany; 2 Cologne Excellence Cluster on Cellular Stress Responses in Aging-Associated Diseases (CECAD), University of Cologne, Germany; 3 German Centre for Infection Research (DZIF), partner site Bonn, Cologne, Germany; 4 Department of Oncohematology, Comenius University and National Cancer Institute, Bratislava, Slovakia

The European Hematology Association (EHA) Guidelines Committee agreed to review the joint global guideline for the diagnosis and management of rare yeast infections of the European Confederation of Medical Mycology (ECMM) in cooperation with the International Society for Human and Animal Mycology (ISHAM) and the American Society for Microbiology (ASM).^1^ External independent referees were nominated and revised the guideline according to a standardized reviewing process; subsequently EHA decided to endorse this guideline into its growing guideline portfolio. Earlier this year, the ECMM-ISHAM-ASM guideline on rare mold infections had already been endorsed by EHA.^2^

Patient numbers among populations at risk for invasive yeast infections are increasing in the clinical routine, such as patients treated on the intensive care unit, those with hematological malignancies or other immunosuppressed and seriously ill patients. Infections due to *Candida* spp. or cryptococcosis have been observed for a long time in this population. However, a growing number of infections due to other emerging yeast species have been described ultimately while to date, their epidemiology remains not well-studied, as with any orphan disease. To make matters worse, management of these diseases poses significant challenges to physicians and morbidity and mortality remains a burden for patients and healthcare systems worldwide. Hence, the necessity to guide microbiologists and clinicians through diagnosis and management of these infections was given.

Clinical guidance on infections due to yeast species other than *Candida* spp. was limited and partially outdated. Furthermore, some pathogenic yeasts were not included at all.^3,4^ The ECMM-ISHAM-ASM guideline was developed by medical professionals from 24 countries analyzing the available evidence body and involving personal expertise in the field. It displays a multidisciplinary, resource-overstretching approach that has been developed within the ECMM global guideline initiative for all fungal entities.^5,6^ The considered pathogens included the genera *Geotrichum, Saprochaete/Magnusiomyces*, and *Trichosporon* species as well as *Kodamaea*, *Malassezia, Pseudozyma* (*Moesziomyces/Dirkmeia), Rhodotorula, Saccharomyces*, and *Sporobolomyces* species.^1^ Detailed recommendations to diagnose these infections including imaging, histopathology, and microbiological techniques as well as antifungal susceptibility testing were given. For treatment, the optimal antifungal therapy was discussed, but also removal of indwelling catheters and surgical approaches, for example, in the case of fungal endocarditis were included. Most of the recommendations are based on scarce evidence or are consensus statements and therefore recommended with moderate to marginal strength. Furthermore, the guideline addressed the issue of recent changes of nomenclature among yeast species, a confounding aspect for clinicians not dealing with these pathogens on an everyday basis. Differences in local epidemiology were also discussed since limited availability of diagnostic tools as well as lacking awareness may hamper the understanding of the true epidemiology of rare yeast infections.

The endorsement by the EHA Guidelines Committee underlines the particular importance of fungal infections in patients with hematological malignancies and those undergoing hematopoietic stem cell transplantation.^7,8^ Despite low absolute patient numbers affected by systemic rare yeast infections, the hematological population is at highest risk for mortality and most often affected by severe disseminated disease. For example, geotrichosis affects mostly hematological patients leading to bloodstream or pulmonary infection with a mortality of up to 65%.^9^ Infections due to *Saprochaete/Magnusiomyces* spp. cause fungemia and subsequently disseminated disease.^8^ Echinocandin monotherapy is contra-intuitively often associated with a worse outcome and treatment regimens including amphotericin b formulations in combination with flucytosine may be required. In trichosporonosis, hematology patients present most frequently with fungemia and/or metastastic skin lesions or pneumonia and hepatosplenic abscesses with voriconazole being the antifungal agent of first choice.^10^

Diagnosis requires first a clinical suspicion and knowledge about risk factors and epidemiology, then a full set of adequate diagnostic mycological methods and ultimately a fast and targeted treatment approach underlining the multiple challenges for treating physicians. This knowledge gap may be closed by the ECMM-ISHAM-ASM guideline on rare yeasts and subsequently improve patient prognosis.^1^ Growing resistance due to widespread use of antifungals, for example, in agriculture and improved prophylactic regimens for invasive fungal infections in at-risk populations may lead to a selection of rare and resistant yeast strains as causative agents in the future.^11^

The ECMM-ISHAM-ASM guideline on rare yeast infections certainly has the intrinsic limitations that recommendations are mostly consensus-based and retrieved evidence is from retrospective data or case studies, an obvious obstacle for all orphan diseases. Due to the general low incidence and heterogeneity of emerging yeast infections, high quality evidence from prospective clinical trials is lacking. Therefore, global databases to gather larger patient cohorts are of high importance in studying rare infections.^12^ With the present ECMM-ISHAM-ASM guideline, an unprecedented effort with a multidisciplinary approach was undertaken to assemble a valuable tool and support clinical decision making. Regional differences in availability of diagnostics or treatment are incorporated to make the recommendations also applicable in lower resource settings. Therefore, this guideline displays a major support to facilitate diagnosis and treatment of rare yeast infections for physicians all over the world with the aim to improve patient management and outcome.

The endorsement by the EHA Guidelines Committee is another step to emphasize the clinical impact of yeast infections in patients with hematological diseases and encourages an interdisciplinary, intersocietal approach for the development of future guidelines for the management of complex diseases.

## Disclosures

JS has received research grants by the Ministry of Education and Research (BMBF) and Basilea Pharmaceuticals Inc., has received speaker honoraria by Pfizer Inc., and has received travel grants by German Society for Infectious Diseases (DGI e.V.) and Meta-Alexander Foundation. LD has received speaker honoraria by Pfizer Inc., Sandoz and Teva Pharmaceuticals.

## References

[1] Chen SC Perfect J Colombo AL. Global guideline for the diagnosis and management of rare yeast infections: an initiative of the ECMM in cooperation with ISHAM and ASM. Lancet Infect Dis. 2021 August 19. [Epub ahead of print]. 10.1016/S1473-3099(21)00203-6 34419208

[2] Sprute R Seidel D Cornely OA. EHA Endorsement of the Global Guideline for the Diagnosis and Management of Rare Mold Infection: An Initiative of the European Confederation of Medical Mycology in Cooperation With International Society for Human and Animal Mycology and American Society for Microbiology. HemaSphere. 2021;5:e519. 33644696 10.1097/HS9.0000000000000519 PMC7904275

[3] Arendrup MC Boekhout T Akova M. ESCMID and ECMM joint clinical guidelines for the diagnosis and management of rare invasive yeast infections. Clin Microbiol Infect. 2014;20(suppl 3):76–98. 10.1111/1469-0691.12360 24102785

[4] Kung HC Huang PY Chen WT. 2016 guidelines for the use of antifungal agents in patients with invasive fungal diseases in Taiwan. J Microbiol Immunol Infect. 2018;51:1–17. 28781150 10.1016/j.jmii.2017.07.006

[5] Cornely OA Lass-Flörl C Lagrou K. Improving outcome of fungal diseases - Guiding experts and patients towards excellence. Mycoses. 2017;60:420–425. 28497502 10.1111/myc.12628

[6] Hoenigl M Gangneux JP Segal E. Global guidelines and initiatives from the European Confederation of Medical Mycology to improve patient care and research worldwide: new leadership is about working together. Mycoses. 2018;61:885–894. 30086186 10.1111/myc.12836

[7] Posteraro B De Carolis E Criscuolo M. Candidaemia in haematological malignancy patients from a SEIFEM study: epidemiological patterns according to antifungal prophylaxis. Mycoses. 2020;63:900–910. 32531854 10.1111/myc.13130

[8] Alp S Gulmez D Ayaz CM. Fungaemia due to rare yeasts in a tertiary care university centre within 18 years. Mycoses. 2020;63:488–493. 32145101 10.1111/myc.13072

[9] Durán Graeff L Seidel D Vehreschild MJ. Invasive infections due to Saprochaete and Geotrichum species: report of 23 cases from the FungiScope Registry. Mycoses. 2017;60:273–279. 28150341 10.1111/myc.12595

[10] de Almeida Júnior JN Hennequin C. Invasive trichosporon infection: a systematic review on a re-emerging fungal pathogen. Front Microbiol. 2016;7:1629. 27799926 10.3389/fmicb.2016.01629 PMC5065970

[11] Pfaller MA Diekema DJ Gibbs DL. Results from the ARTEMIS DISK Global Antifungal Surveillance Study, 1997 to 2007: 10.5-year analysis of susceptibilities of noncandidal yeast species to fluconazole and voriconazole determined by CLSI standardized disk diffusion testing. J Clin Microbiol. 2009;47:117–123. 19005141 10.1128/JCM.01747-08 PMC2620874

[12] Seidel D Durán Graeff LA Vehreschild MJGT. FungiScope™ -global emerging fungal infection registry. Mycoses. 2017;60:508–516. 28730644 10.1111/myc.12631

